# Differences in the Nervous Organisation of Man and Woman: Physiological and Pathological

**Published:** 1893-06

**Authors:** 


*Differences in the Nervous Organisation of Man and Woman: Physiological and Pathological.* By HARRY CAMPBELL, M.D. Pp. xi., 383. London: H. K. LEWIS. 1891.

The author of this work has set himself a difficult and complex task. The subject is of interest to all, but especially to those who have a taste for psychology and for the abstruse problems of heredity; and the scientific aspects of the question are treated with great care and in as thorough a manner as possible. In the first part of the book the theories of Weismann and Geddes are discussed and their investigations and suppositions criticised. With some of these criticisms it is difficult to agree, but Dr. Campbell shows that he has sufficient knowledge of the subject to vindicate his right to form opinions of his own.

The relative proclivity of the two sexes to disease is dealt with at some length. This necessitates a quantity of statistics, which might perhaps have been placed in an appendix, and the deductions from them alone left in the text. The conclusions arrived at seem justified from a study of the figures, but too little importance is attached, we think, to accident, drink, and syphilis in accounting for the greater mortality of the male sex. Many, too, would deny that "man varies between wider extremes than woman," and would rather accept Tennyson's opinion, that

> "Men at most differ as Heaven and Earth,
> But women, worst and best as Heaven and Hell."

Quotations abound, and add greatly to the interest of the book; but we are surprised to see another mistaken rendering of the famous lines from *Hudibras*—

> "He who complies against his will
> Is of the same opinion still,"

which is quoted "*Woman convinced* against her will," &c.

The third part of the book, "Psycho-Physiological," is as clear as such subjects can be made in a comparatively short space. There is little that is original in this part, and the deductions are meagre; but the description of the varieties of weak will is interesting, and the last chapter contains some good but rather trite advice.

Much trouble has been taken with this work; varied and extensive reading must have been necessary, and there is abundant evidence of careful thought and reasoning. The different mental characteristics of man and woman cannot be altogether explained by learning and scientific investigation. Personal observation throughout many years is requisite as well. Here, however, is an important contribution towards a better knowledge of the subject.

